# Magnetoencephalography Studies of the Envelope Following Response During Amplitude-Modulated Sweeps: Diminished Phase Synchrony in Autism Spectrum Disorder

**DOI:** 10.3389/fnhum.2021.787229

**Published:** 2021-12-15

**Authors:** Timothy P. L. Roberts, Luke Bloy, Song Liu, Matthew Ku, Lisa Blaskey, Carissa Jackel

**Affiliations:** ^1^Department of Radiology, Children’s Hospital of Philadelphia, Philadelphia, PA, United States; ^2^Department of Pediatrics, Children’s Hospital of Philadelphia, Philadelphia, PA, United States

**Keywords:** autism (ASD), magnetoencephalography (MEG), gamma-band activity, envelope following response, children

## Abstract

Prevailing theories of the neural basis of at least a subset of individuals with autism spectrum disorder (ASD) include an imbalance of excitatory and inhibitory neurotransmission. These circuitry imbalances are commonly probed in adults using auditory steady-state responses (ASSR, driven at 40 Hz) to elicit coherent electrophysiological responses (EEG/MEG) from intact circuitry. Challenges to the ASSR methodology occur during development, where the optimal ASSR driving frequency may be unknown. An alternative approach (more agnostic to driving frequency) is the amplitude-modulated (AM) sweep in which the amplitude of a tone (with carrier frequency 500 Hz) is modulated as a sweep from 10 to 100 Hz over the course of ∼15 s. Phase synchrony of evoked responses, measured *via* intra-trial coherence, is recorded (by EEG or MEG) as a function of frequency. We applied such AM sweep stimuli bilaterally to 40 typically developing and 80 children with ASD, aged 6–18 years. Diagnoses were confirmed by DSM-5 criteria as well as autism diagnostic observation schedule (ADOS) observational assessment. Stimuli were presented binaurally during MEG recording and consisted of 20 AM swept stimuli (500 Hz carrier; sweep 10–100 Hz up and down) with a duration of ∼30 s each. Peak intra-trial coherence values and peak response frequencies of source modeled responses (auditory cortex) were examined. First, the phase synchrony or inter-trial coherence (ITC) of the ASSR is diminished in ASD; second, hemispheric bias in the ASSR, observed in typical development (TD), is maintained in ASD, and third, that the frequency at which the peak response is obtained varies on an individual basis, in part dependent on age, and with altered developmental trajectories in ASD vs. TD. Finally, there appears an association between auditory steady-state phase synchrony (taken as a proxy of neuronal circuitry integrity) and clinical assessment of language ability/impairment. We concluded that (1) the AM sweep stimulus provides a mechanism for probing ASSR in an unbiased fashion, during developmental maturation of peak response frequency, (2) peak frequencies vary, in part due to developmental age, and importantly, (3) ITC at this peak frequency is diminished in ASD, with the degree of ITC disturbance related to clinically assessed language impairment.

## Introduction

Gamma-band (30–80 Hz) electrophysiological activity elicited from sensory cortices may provide a window into the maturity and viability of local neuronal circuitry. This in turn may be reflective of the balance between excitatory and inhibitory neuronal activity and, as such, the balance of excitatory and inhibitory neurotransmitters (particularly glutamate and GABA). The excitation/inhibition (E/I) imbalance theory has been suggested to be relevant to at least some individuals with ASD for nearly two decades (Rubenstein and Merzenich, 2003; Port et al., 2019; Sohal and Rubenstein, 2019). Thus, considerable attention has been paid to using gamma-band activity as a biomarker, for diagnostic, prognostic, or stratification use.

Eliciting gamma-band activity can be achieved through a variety of stimulation paradigms. The most prevalent paradigm for auditory cortex gamma activity is the auditory steady-state response (ASSR). The ASSR captures the entrainment of auditory cortex neural activity (Pantev et al., 1996; Gutschalk et al., 1999; Brugge et al., 2009; Weisz and Lithari, 2017) to the frequency and phase of auditory stimuli typically delivered as click trains or amplitude-modulated (AM) tones. Studies in adults have suggested that the ASSR is largest in response to presentations in the gamma range particularly at 40 Hz (Galambos et al., 1981; Picton et al., 2003). While the 40 Hz ASSR has been used to illustrate consistent gamma abnormalities in several neurodevelopmental disorders, principally schizophrenia (Brenner et al., 2003; Hong et al., 2004; Light et al., 2006; Hamm et al., 2011; Lenz et al., 2011; Edgar et al., 2014), studies focused on ASD have been more inconsistent (Rojas et al., 2006; Wilson et al., 2007; Edgar et al., 2016; Ono et al., 2020; Seymour et al., 2020; Stroganova et al., 2020).

Initial work examining the ASSR in ASD observed reduced 40 Hz ASSR in children and adolescents (7–17 years) diagnosed with ASD (Wilson et al., 2007) and also in adults (>30 years) diagnosed with ASD and their first-degree relatives (Rojas et al., 2008). A later study, focused on adolescents and early adulthood (14–20 years), similarly found reduced 40 Hz ASSR in participants with ASD (Seymour et al., 2020). Despite the consistent observation of reduced ASSR in adolescent and older populations, studies focused on younger participants have not observed such differences. Examining a large cohort of 7–15-year-old boys, Edgar et al. (2016) did not reveal group differences in ASSR, though bilateral associations with age were observed, as was a right hemispheric dominance of the ASSR responses. Similar age associations and lack of significant group differences have been reported in 7–12-year-old boys (Stroganova et al., 2020) and in 5–7-year-old participants (Ono et al., 2020). Given that the auditory system is still developing through early adulthood, with the 40 Hz ASSR reaching its maximum around 22 years of age (Cho et al., 2015), it may not be surprising that atypical ASSR in neurodevelopmental disorders, such as ASD, do not manifest until later in their maturation (De Stefano et al., 2019).

One possible limitation to previous studies using 40 Hz modulated stimuli in younger children is the question of “optimum” driving frequency for eliciting an ASSR response. While 40 Hz seems canonical for adults, inter-individual variation, especially as a function of development, may compromise the efficiency of a strictly 40 Hz focus. To address this, the “envelope following response (EFR)” has been proposed (Artieda et al., 2004) in which a steady-state stimulus slowly undergoes swept increase of its modulation frequency (e.g., from 10 to 100 Hz, over the course of ∼15 s). Such a stimulus provides a constant “steady-state” stimulus while allowing identification of an “optimal” driving frequency (ascertained by maximum evoked phase coherence), which may occur at 40 Hz, or a frequency slightly lower or higher.

This study examines the neural response to an AM sinusoidal 500 Hz carrier tone. The frequency of the amplitude modulation was continuously swept from 10 to 100 Hz and then back from 100 to 10 Hz over the course of a 30-s stimulus. This approach allowed the peak phase synchrony, or inter-trial coherence (ITC), for each participant to be determined optimally (and not at an arbitrarily predefined frequency) and examined further. In both ASD and typical development (TD) groups, we hypothesized a moderate maturation of peak ASSR frequency over the age range of 6--18 years. More generally, we hypothesized that individuals with ASD^1^ would demonstrate diminished “peak” ITC, reflecting compromised integrity (or delayed maturation) of the local neuronal circuitry of the auditory cortex. Furthermore, we hypothesized that compromise of such neuronal circuitry would have behavioral sequelae in assessments of language ability/impairment.

## Materials and Methods

A total of 80 children with ASD and 40 age-matched TD controls participated in this study. The age range included spanned 6–18 years – refer to Table 1 for demographic information and degree of matching. All studies were approved by the institutional IRB, and written consent was obtained from parents/caregivers, while assent was obtained from participants, over the age of 7 years, who were competent to provide it.

**TABLE 1:** Demographic information.

	**TD**	**ASD**
*N*	40 (6F)	80 (12F)
Handedness	34R, 6L, 0A	69R, 8L, 3A
Age	11.94 ± 0.44	11.72 ± 0.26
NVIQ	**113.24 ± 2.23**	**100.84 ± 1.85**
Equivalent FSIQ (WISC GAI/DAS-II GCA)	**115.32 ± 2.53**	**97.92 ± 2.13**
CELF-CLSS	**108.79 ± 2.00**	**93.36 ± 2.14**
SCQ	**2.63 ± 0.4**	**18.53 ± 0.68**
SRS (T-score)	**42.84 ± 0.72**	**73.44 ± 1.32**
ADOS-CSS	**1.32 ± 0.13**	**6.99 ± 0.2**

*Bold indicates significant differences.*

All participants with ASD had an existing ASD diagnosis, made according to DSM criteria by expert clinicians in the autism specialty practices of medical centers in a large metropolitan area. The diagnosis was confirmed at the time of study participation using gold-standard diagnostic tools, including the Autism Diagnostic Observation Schedule (ADOS-2; Lord et al., 2012) and parent report on the Social Communication Questionnaire (SCQ; Rutter et al., 2003). Dimensional symptom severity ratings were obtained *via* the ADOS-2 Calibrated Severity Score (CSS; Gotham et al., 2009) and parent report on the Social Responsiveness Scale (SRS-2; Constantino and Gruber, 2012).

Data were collected over an extended time period (∼5 years) during which standard diagnostic tests underwent version (as well as protocol) changes: for both the ASD and TD cohorts, cognitive ability was characterized by indices of non-verbal, verbal, and overall intellectual functioning (Table 1), using the Wechsler Intelligence Scale-Fourth Edition (WISC-IV; Wechsler, 2003), the Wechsler Intelligence Scale-Fifth Edition (WISC-V; Wechsler, 2014), or the Differential Ability Scale-Second Edition (DAS-II; Elliott, 2007), depending on the time of study participation. Psychometrics suggest acceptable index-level correlations (*r* = 0.73–0.93) between these tests, all of which are standardized to an average of 100 and SD of 15 (Kuriakose, 2014). Language understanding and expression were evaluated using the core language standard score (CELF-CLSS) from the Clinical Evaluation of Language Fundamentals (CELF-4/CELF-5; Semel et al., 2006, 2013).

To rule out global cognitive delay in both the TD and ASD groups, participants were required to score at or above the second percentile (SS > 70) on the non-verbal composite score of the cognitive assessment administered. Inclusion criteria for all participants included English as their first language. Inclusion criteria for the children with TD included no significant cognitive impairment (described above), no history of neurodevelopmental or psychiatric conditions (e.g., ADHD, learning or language disorder, and depression/anxiety), and scoring below the cutoffs for ASD on all domains of the ADOS-2 (Lord et al., 2012) as well as on parent questionnaires of ASD symptoms (SCQ; Rutter et al., 2003 and SRS-2; Constantino and Gruber, 2012). Additional exclusion criteria for all participants included known neurological disorders (e.g., cerebral palsy and epilepsy), severe tics, and severe head trauma; sensory (e.g., hearing and visual) impairments (e.g., by parent report/medical records); known genetic conditions with a very high incidence of ASD (e.g., Fragile X syndrome and 22q11 deletion syndrome) and premature birth (earlier than gestation of 34 weeks) or significant birth complications.

### Stimuli

Auditory stimuli consisted of sweeps of 30 s duration in which a 500 Hz sinusoidal tone was AM at a rate that increased uniformly between 10 and 100 Hz over the first 15 s (up trials) and then decreased uniformly from 100 to 10 Hz over the remaining 15 s (Down trials). Of note, 20 such tone sweep pairs were presented with an intervening rest/silence period of 9 s for an overall acquisition time of ∼13 min. Stimuli were presented binaurally at the 45 dB sensation level, SL (after individually determining hearing detection thresholds) using Etymotic ER3A transducers and eartip inserts, after amplification and attenuation using TDT series 3 equipment (Tucker Davis Technologies, FL, United States).

### Magnetoencephalography Recording

Recordings were made using a CTF 275-channel whole head biomagnetometer (CTF, Coquitlam, BC, Canada) at a sample rate of 1,200 Hz/channel, housed in a magnetically shielded room, MSR (Vacuumschmelze, Germany). Synthetic third-order gradiometer noise reduction was employed. To facilitate source analysis, a digitized head shape consisting of three anatomical landmarks (i.e., nasion and left and right preauricular points) as well as an additional 200+ points on the scalp and face was obtained for each participant using the fast-track probe position identification system (Polhemus, Colchester, VT, United States).

### Magnetoencephalography Analysis

Magnetoencephalography data were analyzed using the MNE-Python analysis toolbox (Gramfort et al., 2013, 2014). As *sensor*-based analysis of MEG reflects the superposition of activity from multiple brain regions, fine-grained analysis of the temporal features, such as measures of phase coherence, is optimally performed in the *source* rather than sensor space (Hoechstetter et al., 2004; Srinivasan et al., 2006).

Prior to source localization, independent component analysis (ICA; Winkler et al., 2015; Ablin et al., 2018) was used to detect and remove eye-blink and cardiac artifacts. Sensor waveforms were then filtered (0.1–150 Hz) and epochs defined (-1 to 15 s) relative to the onset of the up and down sweeps, resulting in 20 up and 20 down sweep trial trials. To facilitate source localization, an anatomical (1 mm × 1 mm × 1 mm isotropic resolution) MRI (MPRAGE – TE/TR/TI: 2.87 ms/1,900 ms/1,050 ms) was acquired from each participant. Subject-specific single shell head models were created from the MRI data of each participant. Freesurfer (Fischl, 2012) watershed segmentation was used to identify the inner skull surface, and a single shell boundary element model (BEM) forward model was computed. MRI to MEG co-registration was achieved using three fiducial points and further refined using an iterative closest point registration to align acquired digitized head shape points and outer scalp surface of the subject as defined by MRI. A regional source model, with 46 (23-per hemisphere) regional sources (Glasser et al., 2016), along with the Minimum Norm Estimator (MNE; Wang et al., 1992; Hämäläinen and Ilmoniemi, 1994) was used to estimate total power and ITC (Stapells et al., 1987) separately for the up and down trials. For each participant, the peak ITC value was determined, as was the corresponding AM stimulation frequency for both up and down trials.

Statistical treatment: Linear mixed model (LMM) with fixed effects of group, sweep direction, hemisphere with age as a covariate, and subject as a random effect was performed on ITC at the peak of the ASSR response and on the peak frequency itself. To assess the association between MEG measures and behavioral/clinical assessments of language ability, the CLSS of the CELF was included as a covariate, along with age.

## Results

The ASD vs. TD groups did not differ on age (ASD = 11.72 ± 0.26 years, TD = 11.94 ± 0.44 years, *p* = 0.672), and there was no significant difference in distribution of handedness (ASD = 69R, 8L, 3Ambi; TD = 34R, 6L, 0Ambi; chi-squared *p* = 0.354) or sex (ASD = 68 M, 12 F; TD = 34 M, 6 F; chi-squared *p* = 1.00). As expected, groups did significantly differ (*p* < 0.01) on neuropsychological measures known to be affected in the ASD phenotype, such as language ability (CELF-4/5), full scale and non-verbal-IQ (WISC-IV/V or DAS-II), and ADOS-CSS (Shumway et al., 2012; Table 1).

**FIGURE 1:**
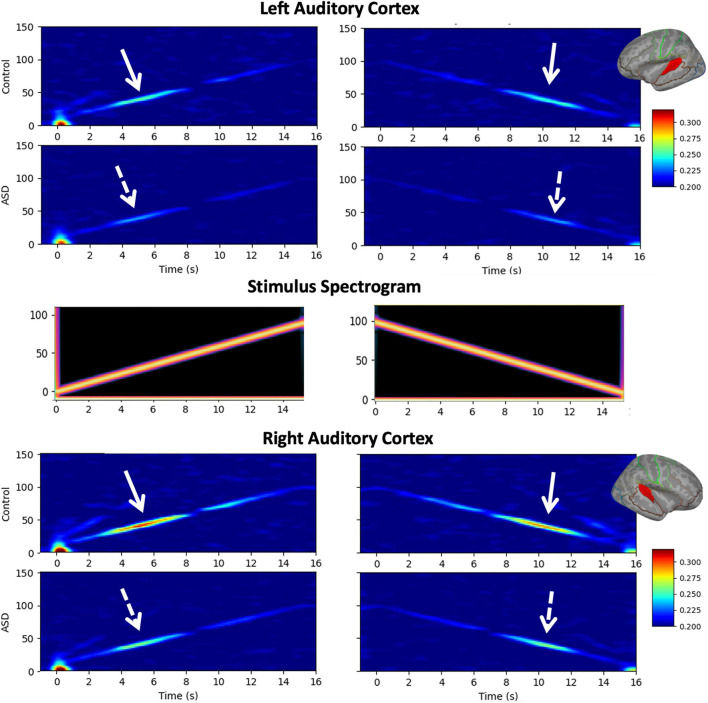
Grand average time-frequency plots [typical development (TD) and autism spectrum disorder (ASD)] of inter-trial coherence (ITC) are shown for the left (**top**) and right (**bottom**) auditory cortices and the up/down sweep directions (**left/right**), as is the spectrogram of the stimuli (**middle**). While increased ITC is visible at all frequencies, the maximal response occurs in the low gamma range (30–50 Hz) where decreased ITC in the ASD group compared to TD is also evident bilaterally, and in both sweep directions.

Figure 1 shows grand average time-frequency ITC responses as a function of time/modulation frequency for both up and down sweeps, for left and right hemispheres, and children with TD vs. ASD. Diminished responses in children with ASD are evident in both hemispheres in the 30–50 Hz range. Notably, in general, left hemispheric responses to seem weaker than right hemispheric responses, while relative group differences between TD and ASD appear to persist across hemispheres.

**FIGURE 2:**
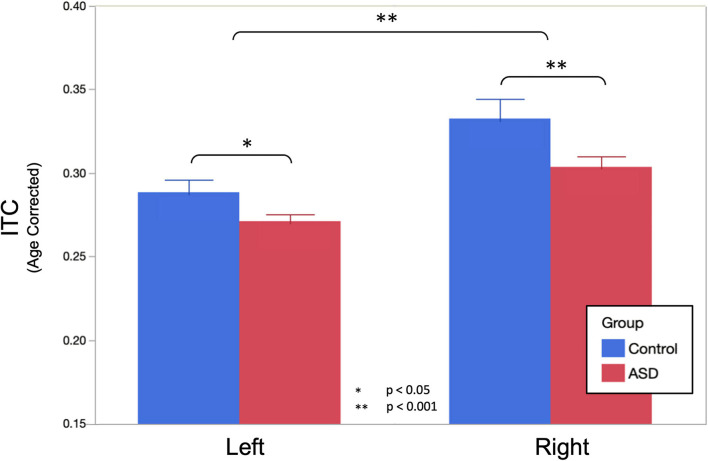
Age corrected auditory steady-state response (ASSR) ITC (to the population mean 11.8 years) at the peak of the ASSR is shown. Omnibus linear mixed models (LMMs) reveal significantly reduced ITC in ASD (main effect of group, *p* = 0.015) and the left hemisphere (main effect of the hemisphere, *p* < 0.001). *Post hoc* analysis in each hemisphere indicated that group differences were more profound in the right hemisphere (*p* < 0.001), compared to the left hemisphere (*p* = 0.029), but significant in both.

A LMM (fixed effects of group, sweep direction, hemisphere with age as a covariate, and subject as a random effect) interrogating the amount of ITC at the peak of the ASSR response revealed significantly reduced ITC in ASD (ASD = 0.288 ± 0.005, TD = 0.311 ± 0.008, *p* = 0.015), a significant difference between hemispheres (LH = 0.280 ± 0.005, RH = 0.318 ± 0.005, *p* < 0.001) as well as a significant group × hemisphere interaction (*p* = 0.045; Figure 2). As expected, no significant effects of sweep direction (or any of its interactions) were observed (up: 0.301 ± 0.005 and down: 0.297 ± 0.005, *p* = 0.15). Significant effects of age (0.120 ± 0.005 per year; *p* < 0.001) as well as age × hemisphere (*p* < 0.001) and age × group (*p* < 0.001) interactions were also observed.

*Post hoc* analysis examining ITC separately in the left and right hemispheres observed that bilateral effects of age were somewhat more profound in the right hemisphere (0.015 ± 0.002 per year, *p* < 0.001) than in the left hemisphere (0.009 ± 0.002 per year, *p* < 0.001). Group differences were also more profound in the right hemisphere (ASD = 0.304 ± 0.007, TD = 0.333 ± 0.010, and *p* < 0.001), compared to the left hemisphere (ASD = 0.272 ± 0.005, TD = 0.289 ± 0.006, *p* = 0.029) as was the interaction between group and age (RH: 0.008 ± 0.002 per year, *p* = 0.0017, LH: 0.005 ± 0.002 per year, *p* = 0.0023).

Similar LMMs revealed no main effect of diagnostic group on peak ASSR response frequency; moreover, when projected to the mean age of 11.8 years, both groups exhibited responses very close to the canonical 40 Hz (ASD = 39.39 ± 0.47 Hz, TD = 40.05 ± 0.66 Hz, and *p* = 0.416). There was also no significant influence of hemisphere on peak ASSR response frequency (LH = 39.99 ± 0.49 Hz, RH = 39.45 ± 0.49 Hz, and *p* = 0.326). Similarly, there was no effect of sweep direction on peak ASSR response frequency (up = 39.86 ± 0.49 Hz, down = 39.58 ± 0.49 Hz, and *p* = 0.609). Although peak ASSR response frequency was very close to 40 Hz at the *projected* mean age of 11.8 years, significant effects of age (0.45 Hz/year and *p* = 0.005) and an age × hemisphere interaction (an additional 0.28 Hz/year in the right hemisphere) were observed.

**FIGURE 3:**
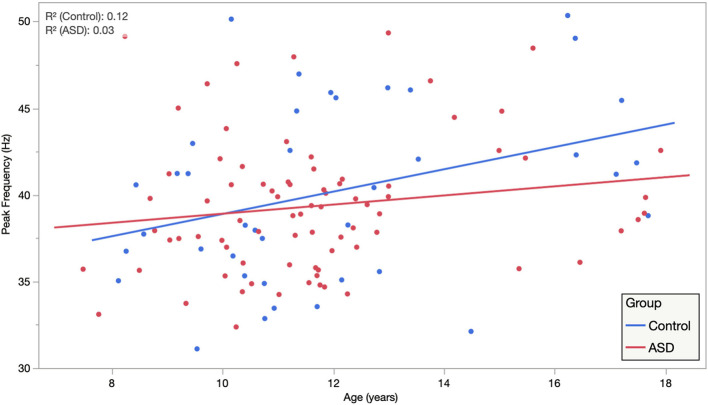
Auditory steady-state response peak ITC frequency, averaged across hemisphere and condition, is plotted against age, illustrating significant age dependence in TD and diminished age dependence in ASD. Importantly, while this suggests that for the younger children a canonical 40 Hz driving frequency stimulus may be suboptimal. It also indicates clear heterogeneity/individuality in the peak ASSR frequency at all ages and possibly also points to an atypical maturational trajectory in ASD as a group.

Based on *a priori* hypotheses of atypical developmental trajectories in participants with ASD (Wolff et al., 2012; Edgar et al., 2015; Berman et al., 2016), the relationship between age and peak ASSR frequency was investigated separately in both groups. Individual LMM models (age, hemisphere, and age × hemisphere) were used in each group, revealing significant age (0.64 Hz/year and *p* = 0.027) and age × hemisphere (additional 0.33 Hz/year in right hemisphere and *p* = 0.0466) effects in TD, while in the ASD group, there was reduced evidence for maturation of peak ASSR frequency (0.26 Hz/year and *p* = 0.15) or age × hemisphere interactions (additional 0.256 Hz/year in right hemisphere and *p* = 0.06). This suggests that for the younger children in the cohort, the use of a canonical 40 Hz driving frequency stimulus would have potentially yielded suboptimal responses (for a 6-year old, the peak ASSR response frequency would be ∼36 Hz), as well as pointing to a group difference in the rate of maturation of the peak response frequency in ASD vs. TD. Figure 3 illustrates the age dependence of peak ASSR response frequency in both TD and ASD.

**FIGURE 4:**
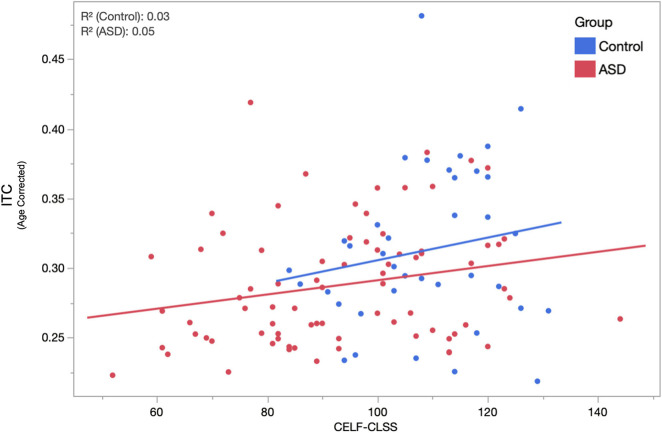
The relationship between age-corrected ASSR ITC (to the population mean 11.8 years, averaged across hemisphere and condition) and assessment of language function (CELF-CLSS) is shown for both ASD and TD groups. Within the ASD group, a significant (*p* = 0.048) positive correlation (increases in ITC indicated increases in language function) was observed. While failing to reach significance (over the narrower range of CELF-CLSS scores encountered), a similar positive relationship is also observed in TD.

Given the high degree of heterogeneity in the language abilities of children diagnosed with ASD and the putative relationship between the ASSR and auditory cortex function, we examined the relationship between peak ASSR ITC value and a behavioral/clinical language ability index: the CLSS of the CELF. LMMs (fixed effects of age, hemisphere, sweep direction, their interactions, and CELF-CLSS with subject as a random effect) were applied separately in the TD and ASD groups. Similar to above, age, hemisphere, and age × hemisphere were significant in both TD and ASD. Additionally, the effect of CELF-CLSS (0.0005 ± 0.0002 and *p* = 0.048) was significant within the ASD group. For visualization purposes (Figure 4), these models were used to project the ITC value to the population mean (11.8 years) and collapsed across the hemisphere and sweep direction (Correlation of ASSR ITC with CELF-CLSS within the ASD cohort – Figure 4).

## Discussion

The main finding of this study is that auditory gamma-band responses, especially in terms of phase synchrony (ITC), are diminished in ASD compared with age-matched TD peers. This is in line with the hypothesis that part of the ASD phenotype stems from an imbalance of excitation and inhibition in the neural circuitry of sensory systems, with such imbalance leading to diminished network capacity to establish coherent electrophysiological oscillatory activity in response to repeated stimuli, with subsequent behavioral/clinical sequelae.

Other findings suggest a developmental trajectory to the optimal driving frequency to elicit ASSR responses such that, especially in younger children, modulation at the canonical 40 Hz may not be optimal for eliciting high signal-to-noise ratio (SNR) responses. Furthermore, the slope of this developmental maturation of ASSR peak frequency appears to be significantly lower in ASD, suggesting either a slower maturation or perhaps the existence of multiple subpopulations encompassing both near-typical developmental trajectories and near-asymptotic ones; only further, longitudinal studies can resolve this on an individual basis.

Moreover, in this large and heterogeneous cohort of children/adolescents with ASD, the peak ASSR ITC (diminished relative to TD peers) was found to correlate with clinical assessment of language impairment, with decreased phase synchrony indicating decreased language performance (with a slope of 0.0005 units of ITC per point on the CELF core language index), suggesting that this electrophysiological signature, putatively mechanistically associated with E/I imbalance, has a clinical/behavioral consequence and thus, meets some of the criteria needed for a diagnostic/prognostic biomarker, at least for the functional domain of language impairment, in ASD.

Given the sample population differences between ASD and TD cohorts in both IQ and language ability, it is not unambiguously possible to ascribe observed differences in brain signals (auditory cortex phase synchrony) to ASD diagnosis alone. It is very likely that such signatures relate to brain activity anomalies shared between ASD and other neuropsychiatric conditions and developmental disorders. Nonetheless, brain signatures of clinical impairment likely point to atypical neuronal circuitry underlying observable behavior. Also, despite similar proportions in both ASD and TD groups, a limitation of this study is the relatively small fraction of female participants, precluding statistical evaluation of sex differences in EFR/ASSR measures.

Additionally, while the main findings of this study might well have been identified with more conventional 40 Hz stimulus paradigms, this study supports the use of the EFR paradigm (a swept-modulation tone), especially in children, to account for individual differences in network maturation and to optimize the collection of signatures of neural synchrony. This might be especially significant for yet younger populations. It is possible that individual differences in optimal ASSR driving frequency might account for some divergent reports on ASSR responsiveness in the literature. It is likely that a narrower frequency sweep (perhaps 30–50 Hz) EFR stimulus might suffice to capture the gamma-band response as well as further define the “resonance width” of the optimal driving frequency.

In summary, electrophysiological responses (particularly, the ITC) elicited by ASSR paradigms reveal anomalies in ASD reflecting the atypical activity of neuronal local circuitry. Furthermore, the magnitude of these anomalies appears to be associated with clinical/behavioral sequelae (namely language impairment). The EFR approach accounts for inter-individual differences in peak ASSR response frequency, attributable to the developmental trajectory or otherwise, and thus, represents a less-biased approach to probing neuronal circuit integrity.

## Data Availability Statement

The raw data supporting the conclusions of this article will be made available by the authors, without undue reservation.

## Ethics Statement

The studies involving human participants were reviewed and approved by Children’s Hospital of Philadelphia Institutional Review Board. Written informed consent to participate in this study was provided by the participants’ legal guardian/next of kin.

## Author Contributions

TR was responsible for the conception of the study, statistical analysis, and wrote the first draft of the manuscript. LuB was responsible for development of data processing pipelines, performed statistical analysis, and edited the manuscript. SL contributed to preprocessing analysis methods and development of figures. MK conducted primary data preprocessing and analysis. LiB conducted neuropsychological assessments and edited the manuscript. CJ provided clinical interpretation on ASD. All authors contributed to the article and approved the submitted version.

## Conflict of Interest

TR declares equity in Prism Clinical Imaging and Proteus Neurodynamics. He serves as a consultant/medical advisory board member for CTF, Ricoh, Spago Nanomedicine, Avexis and Acadia Pharmaceuticals. The remaining authors declare that the research was conducted in the absence of any commercial or financial relationships that could be construed as a potential conflict of interest.

## Publisher’s Note

All claims expressed in this article are solely those of the authors and do not necessarily represent those of their affiliated organizations, or those of the publisher, the editors and the reviewers. Any product that may be evaluated in this article, or claim that may be made by its manufacturer, is not guaranteed or endorsed by the publisher.

## Footnotes

1 Individuals on the autism spectrum, their parents, and professionals in the field have unique and overlapping opinions regarding the use of person-first (e.g., children with ASD) or identity first (e.g., autistic child) language (Kenny et al., 2016). With respect for divided opinions, we used both terms in this study.
